# Supplementary material for: Assessing generalizability of a dengue classifier across multiple datasets
Source: PLoS One. 2025 Jun 3;20(6):e0323886. doi: 10.1371/journal.pone.0323886 (PMC12132959; doi:10.1371/journal.pone.0323886)
Supplement: S1 Table — (PDF) [file pone.0323886.s001.pdf]

Supplementary Table 1. Summary statistics for each dataset.

	Dataset 1 (N=5724)	Dataset 2 (N=1485)	Dataset 3 (N=1552)	Dataset 4, Day -1 (N=257)	Dataset 4, Day -3 (N=257)	Dataset 5 (N=368)
**Dengue**						
Negative	4026 (70.3%)	1018 (68.6%)	1296 (83.5%)	101 (39.3%)	101 (39.3%)	201 (54.6%)
Positive	1698 (29.7%)	467 (31.4%)	256 (16.5%)	156 (60.7%)	156 (60.7%)	167 (45.4%)
**Age**						
Mean (SD)	6.8 (3.6)	25.6 (19.7)	23.6 (17.2)	7.7 (3.1)	7.7 (3.1)	33.0 (11.6)
Median	6.0 (4.0, 9.0)	20.8 (9.1, 37.9)	20.0 (10.0, 33.0)	8.5 (4.5, 10.5)	8.5 (4.5, 10.5)	30.2 (23.8, 39.9)
Range	1.0 - 15.0	1.0 - 98.0	1.0 - 82.0	0.5 - 14.5	0.5 - 14.5	17.0 - 78.4
**White Blood Cell Count (WBC)**						
Mean (SD)	8.6 (4.8)	9.0 (7.0)	10.3 (6.9)	4.2 (2.6)	6.1 (3.9)	7.3 (3.7)
Median	7.5 (5.0, 11.0)	6.9 (4.0, 12.2)	8.7 (5.3, 13.2)	3.4 (2.4, 5.1)	5.1 (3.5, 7.8)	6.7 (4.6, 9.3)
Range	0.9 - 49.6	0.5 - 70.5	0.2 - 47.3	0.6 - 17.9	1.4 - 35.8	1.4 - 21.1
**Platelet Count (PLT)**						
Mean (SD)	231.5 (80.0)	187.0 (123.4)	215.4 (138.8)	173.5 (81.2)	215.6 (80.8)	222.5 (98.6)
Median	224.0 (179.0, 277.0)	160.0 (92.0, 253.0)	194.0 (108.8, 294.0)	166.0 (116.0, 222.0)	205.0 (156.0, 262.0)	222.5 (153.8, 282.8)
Range	18.0 - 829.0	7.0 - 855.0	0.0 - 862.0	18.0 - 427.0	11.0 - 464.0	0.0 - 740.0
