# Supplementary material for: Assessing generalizability of a dengue classifier across multiple datasets
Source: PLoS One. 2025 Jun 3;20(6):e0323886. doi: 10.1371/journal.pone.0323886 (PMC12132959; doi:10.1371/journal.pone.0323886)
Supplement: S2 Table — (PDF) [file pone.0323886.s002.pdf]

## Supplementary Table 2. Full performance metrics for each combination of training/test datasets.

| Training data | Test data | Threshold | Sensitivity | Specificity | PPV | NPV | AUC |
|---|---|---|---|---|---|---|---|
| Dataset 1 | Dataset 1 | Training threshold | 0.747 (0.725, 0.767) | 0.763 (0.75, 0.776) | 0.571 (0.55, 0.591) | 0.877 (0.866, 0.888) | 0.829 (0.817, 0.841) |
| Dataset 1 | Dataset 2 | Training threshold | 0.938 (0.912, 0.958) | 0.296 (0.268, 0.325) | 0.379 (0.351, 0.408) | 0.912 (0.876, 0.94) | 0.596 (0.568, 0.624) |
| Dataset 1 | Dataset 2 | Test threshold | 0.899 (0.868, 0.925) | 0.344 (0.315, 0.374) | 0.386 (0.357, 0.416) | 0.882 (0.846, 0.912) | 0.596 (0.568, 0.624) |
| Dataset 1 | Dataset 3 | Training threshold | 0.914 (0.873, 0.945) | 0.366 (0.339, 0.393) | 0.222 (0.197, 0.248) | 0.956 (0.934, 0.972) | 0.667 (0.638, 0.697) |
| Dataset 1 | Dataset 3 | Test threshold | 0.965 (0.934, 0.984) | 0.297 (0.272, 0.323) | 0.213 (0.19, 0.238) | 0.977 (0.957, 0.99) | 0.667 (0.638, 0.697) |
| Dataset 1 | Dataset 4, Day -3 | Training threshold | 0.827 (0.758, 0.883) | 0.693 (0.593, 0.781) | 0.806 (0.736, 0.864) | 0.722 (0.621, 0.808) | 0.818 (0.763, 0.873) |
| Dataset 1 | Dataset 4, Day -3 | Test threshold | 0.449 (0.369, 0.53) | 0.921 (0.85, 0.965) | 0.897 (0.808, 0.955) | 0.52 (0.444, 0.595) | 0.818 (0.763, 0.873) |
| Dataset 1 | Dataset 4, Day -1 | Training threshold | 0.936 (0.885, 0.969) | 0.446 (0.347, 0.548) | 0.723 (0.656, 0.783) | 0.818 (0.691, 0.909) | 0.868 (0.823, 0.913) |
| Dataset 1 | Dataset 4, Day -1 | Test threshold | 0.705 (0.627, 0.775) | 0.881 (0.802, 0.937) | 0.902 (0.834, 0.948) | 0.659 (0.573, 0.739) | 0.868 (0.823, 0.913) |
| Dataset 1 | Dataset 5 | Training threshold | 0.994 (0.967, 1) | 0.06 (0.031, 0.102) | 0.468 (0.415, 0.521) | 0.923 (0.64, 0.998) | 0.663 (0.607, 0.718) |
| Dataset 1 | Dataset 5 | Test threshold | 0.97 (0.932, 0.99) | 0.095 (0.058, 0.144) | 0.471 (0.417, 0.525) | 0.792 (0.578, 0.929) | 0.663 (0.607, 0.718) |
| Dataset 2 | Dataset 1 | Training threshold | 0.554 (0.53, 0.578) | 0.879 (0.868, 0.889) | 0.659 (0.633, 0.683) | 0.824 (0.812, 0.835) | 0.806 (0.793, 0.818) |
| Dataset 2 | Dataset 1 | Test threshold | 0.689 (0.666, 0.711) | 0.765 (0.752, 0.778) | 0.553 (0.531, 0.574) | 0.854 (0.842, 0.865) | 0.806 (0.793, 0.818) |
| Dataset 2 | Dataset 2 | Training threshold | 0.805 (0.766, 0.84) | 0.847 (0.823, 0.868) | 0.707 (0.666, 0.745) | 0.905 (0.884, 0.922) | 0.895 (0.878, 0.912) |
| Dataset 2 | Dataset 3 | Training threshold | 0.625 (0.563, 0.685) | 0.856 (0.835, 0.874) | 0.461 (0.408, 0.515) | 0.92 (0.904, 0.935) | 0.853 (0.828, 0.877) |
| Dataset 2 | Dataset 3 | Test threshold | 0.84 (0.789, 0.883) | 0.715 (0.69, 0.74) | 0.368 (0.329, 0.409) | 0.958 (0.943, 0.969) | 0.853 (0.828, 0.877) |
| Dataset 2 | Dataset 4, Day -3 | Training threshold | 0.571 (0.489, 0.649) | 0.802 (0.711, 0.875) | 0.817 (0.731, 0.884) | 0.547 (0.463, 0.629) | 0.776 (0.718, 0.835) |
| Dataset 2 | Dataset 4, Day -3 | Test threshold | 0.378 (0.302, 0.459) | 0.911 (0.838, 0.958) | 0.868 (0.764, 0.938) | 0.487 (0.414, 0.56) | 0.776 (0.718, 0.835) |
| Dataset 2 | Dataset 4, Day -1 | Training threshold | 0.891 (0.831, 0.935) | 0.624 (0.522, 0.718) | 0.785 (0.717, 0.843) | 0.787 (0.682, 0.871) | 0.844 (0.796, 0.893) |
| Dataset 2 | Dataset 4, Day -1 | Test threshold | 0.667 (0.587, 0.74) | 0.782 (0.689, 0.858) | 0.825 (0.748, 0.887) | 0.603 (0.514, 0.687) | 0.844 (0.796, 0.893) |
| Dataset 2 | Dataset 5 | Training threshold | 0.335 (0.264, 0.412) | 0.96 (0.923, 0.983) | 0.875 (0.768, 0.944) | 0.635 (0.578, 0.689) | 0.735 (0.683, 0.787) |
| Dataset 2 | Dataset 5 | Test threshold | 0.347 (0.275, 0.425) | 0.945 (0.904, 0.972) | 0.841 (0.733, 0.918) | 0.635 (0.578, 0.69) | 0.735 (0.683, 0.787) |
| Dataset 3 | Dataset 1 | Training threshold | 0.689 (0.666, 0.711) | 0.767 (0.753, 0.78) | 0.555 (0.533, 0.576) | 0.854 (0.842, 0.865) | 0.803 (0.791, 0.815) |
| Dataset 3 | Dataset 1 | Test threshold | 0.438 (0.414, 0.462) | 0.923 (0.914, 0.931) | 0.706 (0.677, 0.733) | 0.796 (0.784, 0.807) | 0.803 (0.791, 0.815) |
| Dataset 3 | Dataset 2 | Training threshold | 0.876 (0.842, 0.904) | 0.744 (0.716, 0.77) | 0.61 (0.572, 0.648) | 0.929 (0.909, 0.946) | 0.894 (0.877, 0.911) |
| Dataset 3 | Dataset 2 | Test threshold | 0.548 (0.502, 0.594) | 0.951 (0.936, 0.963) | 0.837 (0.79, 0.876) | 0.821 (0.798, 0.843) | 0.894 (0.877, 0.911) |
| Dataset 3 | Dataset 3 | Training threshold | 0.781 (0.726, 0.83) | 0.78 (0.757, 0.802) | 0.412 (0.368, 0.458) | 0.948 (0.932, 0.96) | 0.854 (0.829, 0.878) |
| Dataset 3 | Dataset 4, Day -3 | Training threshold | 0.731 (0.654, 0.799) | 0.663 (0.562, 0.754) | 0.77 (0.694, 0.835) | 0.615 (0.517, 0.706) | 0.763 (0.703, 0.823) |
| Dataset 3 | Dataset 4, Day -3 | Test threshold | 0.038 (0.014, 0.082) | 0.98 (0.93, 0.998) | 0.75 (0.349, 0.968) | 0.398 (0.336, 0.461) | 0.763 (0.703, 0.823) |
| Dataset 3 | Dataset 4, Day -1 | Training threshold | 0.923 (0.869, 0.96) | 0.485 (0.384, 0.587) | 0.735 (0.667, 0.795) | 0.803 (0.682, 0.894) | 0.838 (0.789, 0.887) |
| Dataset 3 | Dataset 4, Day -1 | Test threshold | 0.128 (0.08, 0.191) | 0.99 (0.946, 1) | 0.952 (0.762, 0.999) | 0.424 (0.36, 0.49) | 0.838 (0.789, 0.887) |
| Dataset 3 | Dataset 5 | Training threshold | 0.425 (0.349, 0.504) | 0.886 (0.833, 0.926) | 0.755 (0.656, 0.838) | 0.65 (0.59, 0.706) | 0.742 (0.691, 0.793) |
| Dataset 3 | Dataset 5 | Test threshold | 0.114 (0.07, 0.172) | 1 (0.982, 1) | 1 (0.824, 1) | 0.576 (0.522, 0.628) | 0.742 (0.691, 0.793) |
| Dataset 4, Day -3 | Dataset 1 | Training threshold | 0.697 (0.675, 0.719) | 0.793 (0.781, 0.806) | 0.587 (0.565, 0.609) | 0.861 (0.85, 0.872) | 0.821 (0.809, 0.833) |
| Dataset 4, Day -3 | Dataset 1 | Test threshold | 0.856 (0.838, 0.872) | 0.574 (0.558, 0.589) | 0.458 (0.441, 0.476) | 0.904 (0.892, 0.915) | 0.821 (0.809, 0.833) |
| Dataset 4, Day -3 | Dataset 2 | Training threshold | 0.901 (0.871, 0.927) | 0.303 (0.274, 0.332) | 0.372 (0.344, 0.401) | 0.87 (0.831, 0.903) | 0.547 (0.518, 0.575) |
| Dataset 4, Day -3 | Dataset 2 | Test threshold | 0.936 (0.91, 0.956) | 0.266 (0.239, 0.295) | 0.369 (0.342, 0.397) | 0.9 (0.861, 0.932) | 0.547 (0.518, 0.575) |
| Dataset 4, Day -3 | Dataset 3 | Training threshold | 0.906 (0.864, 0.939) | 0.363 (0.337, 0.39) | 0.219 (0.195, 0.246) | 0.952 (0.929, 0.969) | 0.626 (0.596, 0.656) |
| Dataset 4, Day -3 | Dataset 3 | Test threshold | 0.969 (0.939, 0.986) | 0.234 (0.211, 0.258) | 0.2 (0.178, 0.223) | 0.974 (0.95, 0.989) | 0.626 (0.596, 0.656) |
| Dataset 4, Day -3 | Dataset 4, Day -3 | Training threshold | 0.833 (0.765, 0.888) | 0.723 (0.625, 0.807) | 0.823 (0.754, 0.879) | 0.737 (0.639, 0.821) | 0.835 (0.783, 0.888) |
| Dataset 4, Day -3 | Dataset 5 | Training threshold | 0.994 (0.967, 1) | 0.045 (0.021, 0.083) | 0.464 (0.411, 0.517) | 0.9 (0.555, 0.997) | 0.622 (0.565, 0.679) |
| Dataset 4, Day -3 | Dataset 5 | Test threshold | 1 (0.978, 1) | 0.03 (0.011, 0.064) | 0.461 (0.409, 0.514) | 1 (0.541, 1) | 0.622 (0.565, 0.679) |
| Dataset 4, Day -1 | Dataset 1 | Training threshold | 0.423 (0.4, 0.447) | 0.951 (0.944, 0.958) | 0.785 (0.757, 0.811) | 0.796 (0.785, 0.808) | 0.828 (0.816, 0.84) |
| Dataset 4, Day -1 | Dataset 1 | Test threshold | 0.676 (0.653, 0.698) | 0.824 (0.812, 0.835) | 0.618 (0.595, 0.64) | 0.858 (0.846, 0.868) | 0.828 (0.816, 0.84) |
| Dataset 4, Day -1 | Dataset 2 | Training threshold | 0.861 (0.826, 0.891) | 0.509 (0.478, 0.54) | 0.446 (0.413, 0.479) | 0.889 (0.86, 0.913) | 0.693 (0.666, 0.719) |
| Dataset 4, Day -1 | Dataset 2 | Test threshold | 0.895 (0.864, 0.921) | 0.453 (0.422, 0.484) | 0.429 (0.397, 0.46) | 0.904 (0.875, 0.928) | 0.693 (0.666, 0.719) |
| Dataset 4, Day -1 | Dataset 3 | Training threshold | 0.781 (0.726, 0.83) | 0.607 (0.58, 0.634) | 0.282 (0.249, 0.317) | 0.934 (0.915, 0.949) | 0.729 (0.701, 0.758) |
| Dataset 4, Day -1 | Dataset 3 | Test threshold | 0.906 (0.864, 0.939) | 0.448 (0.421, 0.476) | 0.245 (0.218, 0.274) | 0.96 (0.942, 0.974) | 0.729 (0.701, 0.758) |
| Dataset 4, Day -1 | Dataset 4, Day -1 | Training threshold | 0.821 (0.751, 0.877) | 0.762 (0.667, 0.841) | 0.842 (0.774, 0.896) | 0.733 (0.638, 0.815) | 0.874 (0.831, 0.917) |
| Dataset 4, Day -1 | Dataset 5 | Training threshold | 0.838 (0.774, 0.891) | 0.368 (0.301, 0.439) | 0.524 (0.463, 0.586) | 0.733 (0.635, 0.816) | 0.701 (0.648, 0.755) |
| Dataset 4, Day -1 | Dataset 5 | Test threshold | 0.892 (0.835, 0.935) | 0.269 (0.209, 0.336) | 0.503 (0.445, 0.562) | 0.75 (0.634, 0.845) | 0.701 (0.648, 0.755) |
| Dataset 5 | Dataset 1 | Training threshold | 0.783 (0.762, 0.802) | 0.67 (0.656, 0.685) | 0.5 (0.481, 0.52) | 0.88 (0.868, 0.891) | 0.808 (0.796, 0.82) |
| Dataset 5 | Dataset 1 | Test threshold | 0.889 (0.873, 0.904) | 0.479 (0.464, 0.495) | 0.419 (0.402, 0.435) | 0.911 (0.898, 0.923) | 0.808 (0.796, 0.82) |
| Dataset 5 | Dataset 2 | Training threshold | 0.936 (0.91, 0.956) | 0.547 (0.516, 0.578) | 0.487 (0.453, 0.52) | 0.949 (0.928, 0.965) | 0.873 (0.854, 0.892) |
| Dataset 5 | Dataset 2 | Test threshold | 0.936 (0.91, 0.956) | 0.578 (0.547, 0.608) | 0.504 (0.47, 0.538) | 0.951 (0.931, 0.967) | 0.873 (0.854, 0.892) |
| Dataset 5 | Dataset 3 | Training threshold | 0.871 (0.824, 0.91) | 0.593 (0.566, 0.62) | 0.297 (0.265, 0.331) | 0.959 (0.943, 0.972) | 0.839 (0.813, 0.866) |
| Dataset 5 | Dataset 3 | Test threshold | 0.969 (0.939, 0.986) | 0.325 (0.299, 0.351) | 0.221 (0.197, 0.246) | 0.981 (0.964, 0.992) | 0.839 (0.813, 0.866) |
| Dataset 5 | Dataset 4, Day -3 | Training threshold | 0.788 (0.716, 0.85) | 0.594 (0.492, 0.691) | 0.75 (0.677, 0.814) | 0.645 (0.539, 0.742) | 0.761 (0.701, 0.821) |
| Dataset 5 | Dataset 4, Day -3 | Test threshold | 0.442 (0.363, 0.524) | 0.881 (0.802, 0.937) | 0.852 (0.756, 0.921) | 0.506 (0.429, 0.582) | 0.761 (0.701, 0.821) |
| Dataset 5 | Dataset 4, Day -1 | Training threshold | 0.949 (0.901, 0.978) | 0.347 (0.255, 0.448) | 0.692 (0.625, 0.753) | 0.814 (0.666, 0.916) | 0.841 (0.792, 0.889) |
| Dataset 5 | Dataset 4, Day -1 | Test threshold | 0.692 (0.614, 0.764) | 0.802 (0.711, 0.875) | 0.844 (0.769, 0.902) | 0.628 (0.538, 0.711) | 0.841 (0.792, 0.889) |
| Dataset 5 | Dataset 5 | Training threshold | 0.707 (0.631, 0.774) | 0.632 (0.561, 0.699) | 0.615 (0.542, 0.684) | 0.722 (0.649, 0.786) | 0.75 (0.699, 0.801) |
