# Supplementary material for: Assessing generalizability of a dengue classifier across multiple datasets
Source: PLoS One. 2025 Jun 3;20(6):e0323886. doi: 10.1371/journal.pone.0323886 (PMC12132959; doi:10.1371/journal.pone.0323886)
Supplement: S3 Table — (PDF) [file pone.0323886.s003.pdf]

Supplementary Table 3. Label shift adjustment and estimation for each training/test pair.

Training data	Test data	Deviance (uncorrected predictions)	Deviance (corrected predictions)	Training prevalence	Test prevalence	Est. test prevalence, average	Est. test prevalence, discretization	Est. test prevalence, fixed point
Dataset 1	Dataset 2 (Age < 16)	0.90	0.93	0.30	0.37	0.39	0.49	0.68
Dataset 1	Dataset 3 (Age < 16)	0.78	0.67	0.30	0.14	0.26	0.20	0.25
Dataset 1	Dataset 4, Day -1	0.95	1.12	0.30	0.61	0.56	0.99	1.16
Dataset 1	Dataset 4, Day -3	1.20	1.03	0.30	0.61	0.42	0.77	0.73
Dataset 1	Dataset 5	2.79	3.35	0.30	0.45	0.84	0.99	1.79
Dataset 2	Dataset 1	0.97	0.97	0.31	0.30	0.28	0.17	0.13
Dataset 2	Dataset 3	0.71	0.68	0.31	0.17	0.23	0.10	0.12
Dataset 2	Dataset 4, Day -1	0.98	1.14	0.31	0.61	0.57	0.97	0.85
Dataset 2	Dataset 4, Day -3	1.35	1.10	0.31	0.61	0.39	0.62	0.39
Dataset 2	Dataset 5	1.75	1.51	0.31	0.45	0.21	0.01	0.04
Dataset 3	Dataset 1	1.06	0.97	0.17	0.30	0.18	0.30	0.03
Dataset 3	Dataset 2	0.84	0.79	0.17	0.31	0.23	0.41	0.43
Dataset 3	Dataset 4, Day -1	1.25	1.18	0.17	0.61	0.39	0.99	1.09
Dataset 3	Dataset 4, Day -3	1.77	1.12	0.17	0.61	0.26	0.77	0.18
Dataset 3	Dataset 5	1.92	1.30	0.17	0.45	0.15	0.06	-0.05
Dataset 4, Day -3	Dataset 1	1.05	0.96	0.61	0.30	0.42	0.14	0.11
Dataset 4, Day -3	Dataset 2 (Age < 16)	1.07	0.98	0.61	0.37	0.47	0.35	0.27
Dataset 4, Day -3	Dataset 3 (Age < 16)	1.20	0.74	0.61	0.14	0.37	0.12	0.02
Dataset 4, Day -3	Dataset 5	5.33	4.72	0.61	0.45	0.95	0.99	1.20
Dataset 4, Day -1	Dataset 1	0.99	1.26	0.61	0.30	0.26	0.01	-0.15
Dataset 4, Day -1	Dataset 2 (Age < 16)	1.02	1.07	0.61	0.37	0.38	0.23	0.14
Dataset 4, Day -1	Dataset 3 (Age < 16)	0.88	0.87	0.61	0.14	0.22	0.01	-0.20
Dataset 4, Day -1	Dataset 5	2.63	2.29	0.61	0.45	0.75	0.85	0.83
Dataset 5	Dataset 1	1.04	0.98	0.45	0.30	0.40	0.10	0.26
Dataset 5	Dataset 2	1.05	0.91	0.45	0.31	0.49	0.57	0.77
Dataset 5	Dataset 3	1.06	0.69	0.45	0.17	0.41	0.29	0.42
Dataset 5	Dataset 4, Day -1	1.05	1.12	0.45	0.61	0.60	0.99	1.28
Dataset 5	Dataset 4, Day -3	1.20	1.14	0.45	0.61	0.49	0.69	0.78
