# Supplementary material for: Assessing generalizability of a dengue classifier across multiple datasets
Source: PLoS One. 2025 Jun 3;20(6):e0323886. doi: 10.1371/journal.pone.0323886 (PMC12132959; doi:10.1371/journal.pone.0323886)
Supplement: S4 Table — Model denotes whether the model evaluated is the original model (age, WBC, and PLT), the full model (using all available variables in the subset), or the model chosen by best subsets selection (BSS) with that training data. Missing values in the PPV indicate that there were 0 predicted positive cases. (PDF) [file pone.0323886.s004.pdf]

Supplementary Table 4. In-sample and generalizability performance metrics for logistic regression with each training/test pair, Alternative Subset 1. Model denotes whether the model evaluated is the original model (age, WBC, and PLT), the full model (using all available variables in the subset), or the model chosen by best subsets selection (BSS) with that training data. Missing values in the PPV indicate that there were 0 predicted positive cases.

Training data	Test data	Model	Sensitivity	Specificity	PPV	NPV	AUC
Dataset 1	Dataset 1	Original	0.742 (0.721, 0.763)	0.757 (0.743, 0.77)	0.563 (0.542, 0.583)	0.874 (0.863, 0.885)	0.83 (0.81, 0.84)
Dataset 1	Dataset 1	Full	0.767 (0.747, 0.787)	0.775 (0.762, 0.788)	0.59 (0.57, 0.611)	0.888 (0.877, 0.898)	0.85 (0.84, 0.86)
Dataset 1	Dataset 1	BSS	0.767 (0.747, 0.787)	0.778 (0.765, 0.791)	0.593 (0.572, 0.614)	0.888 (0.877, 0.898)	0.85 (0.84, 0.86)
Dataset 1	Dataset 3	Original	0.914 (0.873, 0.945)	0.367 (0.341, 0.395)	0.228 (0.203, 0.255)	0.954 (0.932, 0.971)	0.669 (0.639, 0.698)
Dataset 1	Dataset 3	Full	0 (0, 0.014)	0.999 (0.996, 1)	0 (0, 0.975)	0.83 (0.81, 0.849)	0.679 (0.652, 0.706)
Dataset 1	Dataset 3	BSS	0 (0, 0.014)	0.999 (0.996, 1)	0 (0, 0.975)	0.83 (0.81, 0.849)	0.693 (0.665, 0.722)
Dataset 1	Dataset 3 (Age < 16)	Original	0.759 (0.653, 0.846)	0.751 (0.709, 0.789)	0.348 (0.279, 0.422)	0.947 (0.919, 0.967)	0.83 (0.782, 0.879)
Dataset 1	Dataset 3 (Age < 16)	Full	0 (0, 0.043)	1 (0.992, 1)		0.851 (0.818, 0.879)	0.434 (0.381, 0.487)
Dataset 1	Dataset 3 (Age < 16)	BSS	0 (0, 0.043)	1 (0.992, 1)		0.851 (0.818, 0.879)	0.425 (0.37, 0.48)
Dataset 1	Dataset 5	Original	0.994 (0.967, 1)	0.062 (0.032, 0.105)	0.474 (0.421, 0.528)	0.923 (0.64, 0.998)	0.67 (0.614, 0.726)
Dataset 1	Dataset 5	Full	0 (0, 0.022)	1 (0.981, 1)		0.54 (0.487, 0.592)	0.474 (0.428, 0.52)
Dataset 1	Dataset 5	BSS	0 (0, 0.022)	1 (0.981, 1)		0.54 (0.487, 0.592)	0.463 (0.413, 0.514)
Dataset 3	Dataset 3	Original	0.789 (0.734, 0.837)	0.776 (0.751, 0.798)	0.418 (0.374, 0.464)	0.947 (0.932, 0.96)	0.85 (0.82, 0.87)
Dataset 3	Dataset 3	Full	0.773 (0.717, 0.823)	0.801 (0.778, 0.823)	0.443 (0.396, 0.49)	0.945 (0.93, 0.958)	0.87 (0.84, 0.89)
Dataset 3	Dataset 3	BSS	0.773 (0.717, 0.823)	0.8 (0.776, 0.821)	0.441 (0.394, 0.488)	0.945 (0.93, 0.958)	0.87 (0.84, 0.89)
Dataset 3	Dataset 1	Original	0.695 (0.672, 0.717)	0.756 (0.743, 0.769)	0.546 (0.525, 0.567)	0.855 (0.843, 0.866)	0.803 (0.79, 0.815)
Dataset 3	Dataset 1	Full	0.959 (0.949, 0.968)	0.253 (0.239, 0.266)	0.351 (0.337, 0.365)	0.936 (0.92, 0.95)	0.805 (0.793, 0.818)
Dataset 3	Dataset 1	BSS	0.942 (0.929, 0.952)	0.328 (0.314, 0.343)	0.372 (0.357, 0.386)	0.93 (0.916, 0.943)	0.813 (0.8, 0.825)
Dataset 3	Dataset 5	Original	0.44 (0.363, 0.519)	0.882 (0.828, 0.924)	0.76 (0.663, 0.842)	0.649 (0.588, 0.706)	0.74 (0.688, 0.792)
Dataset 3	Dataset 5	Full	0.596 (0.518, 0.672)	0.749 (0.682, 0.808)	0.669 (0.587, 0.744)	0.685 (0.618, 0.747)	0.715 (0.661, 0.768)
Dataset 3	Dataset 5	BSS	0.578 (0.499, 0.654)	0.779 (0.715, 0.836)	0.691 (0.607, 0.766)	0.685 (0.619, 0.745)	0.724 (0.67, 0.777)
Dataset 5	Dataset 5	Original	0.693 (0.617, 0.762)	0.641 (0.569, 0.708)	0.622 (0.548, 0.692)	0.71 (0.637, 0.776)	0.73 (0.68, 0.79)
Dataset 5	Dataset 5	Full	0.711 (0.636, 0.778)	0.641 (0.569, 0.708)	0.628 (0.554, 0.697)	0.723 (0.649, 0.788)	0.72 (0.66, 0.77)
Dataset 5	Dataset 5	BSS	0.717 (0.642, 0.784)	0.636 (0.564, 0.703)	0.626 (0.553, 0.695)	0.725 (0.652, 0.791)	0.74 (0.69, 0.79)
Dataset 5	Dataset 1	Original	0.777 (0.756, 0.796)	0.68 (0.665, 0.694)	0.506 (0.486, 0.525)	0.878 (0.866, 0.89)	0.809 (0.797, 0.821)
Dataset 5	Dataset 1	Full	0.852 (0.834, 0.868)	0.526 (0.511, 0.542)	0.431 (0.414, 0.448)	0.894 (0.881, 0.906)	0.8 (0.788, 0.813)
Dataset 5	Dataset 1	BSS	0.753 (0.732, 0.774)	0.675 (0.66, 0.689)	0.494 (0.475, 0.514)	0.866 (0.854, 0.878)	0.786 (0.774, 0.799)
Dataset 5	Dataset 3	Original	0.863 (0.815, 0.903)	0.593 (0.565, 0.62)	0.302 (0.269, 0.337)	0.955 (0.938, 0.968)	0.836 (0.81, 0.863)
Dataset 5	Dataset 3	Full	0.859 (0.811, 0.9)	0.597 (0.57, 0.625)	0.304 (0.271, 0.339)	0.954 (0.937, 0.968)	0.824 (0.796, 0.853)
Dataset 5	Dataset 3	BSS	0.875 (0.828, 0.913)	0.598 (0.57, 0.626)	0.308 (0.275, 0.343)	0.959 (0.943, 0.972)	0.837 (0.81, 0.864)
Dataset 5	Dataset 3 (Age > 16)	Original	0.939 (0.89, 0.97)	0.522 (0.486, 0.558)	0.298 (0.258, 0.339)	0.975 (0.955, 0.988)	0.861 (0.832, 0.89)
Dataset 5	Dataset 3 (Age > 16)	Full	0.957 (0.914, 0.983)	0.527 (0.491, 0.563)	0.304 (0.265, 0.346)	0.983 (0.965, 0.993)	0.867 (0.839, 0.896)
Dataset 5	Dataset 3 (Age > 16)	BSS	0.945 (0.898, 0.974)	0.574 (0.537, 0.609)	0.324 (0.282, 0.368)	0.98 (0.962, 0.991)	0.884 (0.857, 0.912)
