# Supplementary material for: Assessing generalizability of a dengue classifier across multiple datasets
Source: PLoS One. 2025 Jun 3;20(6):e0323886. doi: 10.1371/journal.pone.0323886 (PMC12132959; doi:10.1371/journal.pone.0323886)
Supplement: S6 Table — Model denotes whether the model evaluated is the original model (age, WBC, and PLT), the full model (using all available variables in the subset), or the model chosen by best subsets selection (BSS) with that training data. (PDF) [file pone.0323886.s006.pdf]

Supplementary Table 6. In-sample and generalizability performance metrics for logistic regression with each training/test pair, Alternative Subset 3. Model denotes whether the model evaluated is the original model (age, WBC, and PLT), the full model (using all available variables in the subset), or the model chosen by best subsets selection (BSS) with that training data.

Training data	Test data	Model	Sensitivity	Specificity	PPV	NPV	AUC
Dataset 1	Dataset 1	Original	0.743 (0.722, 0.764)	0.757 (0.744, 0.77)	0.563 (0.543, 0.584)	0.875 (0.864, 0.886)	0.83 (0.81, 0.84)
Dataset 1	Dataset 1	Full	0.778 (0.757, 0.797)	0.786 (0.773, 0.798)	0.605 (0.584, 0.625)	0.893 (0.883, 0.903)	0.86 (0.85, 0.87)
Dataset 1	Dataset 1	BSS	0.779 (0.758, 0.799)	0.785 (0.772, 0.797)	0.604 (0.583, 0.624)	0.894 (0.883, 0.904)	0.86 (0.85, 0.87)
Dataset 1	Dataset 3	Original	0.961 (0.912, 0.987)	0.262 (0.23, 0.296)	0.187 (0.158, 0.219)	0.974 (0.941, 0.992)	0.643 (0.604, 0.683)
Dataset 1	Dataset 3	Full	0.016 (0.002, 0.055)	0.877 (0.85, 0.9)	0.022 (0.003, 0.076)	0.834 (0.806, 0.86)	0.801 (0.758, 0.845)
Dataset 1	Dataset 3	BSS	0.023 (0.005, 0.066)	0.872 (0.846, 0.896)	0.031 (0.006, 0.089)	0.835 (0.806, 0.86)	0.799 (0.755, 0.843)
Dataset 1	Dataset 3 (Age < 16)	Original	0.792 (0.578, 0.929)	0.747 (0.674, 0.811)	0.311 (0.199, 0.443)	0.961 (0.912, 0.987)	0.86 (0.783, 0.937)
Dataset 1	Dataset 3 (Age < 16)	Full	0 (0, 0.142)	0.97 (0.931, 0.99)	0 (0, 0.522)	0.87 (0.813, 0.915)	0.697 (0.571, 0.824)
Dataset 1	Dataset 3 (Age < 16)	BSS	0 (0, 0.142)	0.97 (0.931, 0.99)	0 (0, 0.522)	0.87 (0.813, 0.915)	0.695 (0.569, 0.822)
Dataset 1	Dataset 4, Day -3	Original	0.827 (0.758, 0.883)	0.693 (0.593, 0.781)	0.806 (0.736, 0.864)	0.722 (0.621, 0.808)	0.818 (0.763, 0.873)
Dataset 1	Dataset 4, Day -3	Full	0.006 (0, 0.035)	1 (0.964, 1)	1 (0.025, 1)	0.395 (0.334, 0.457)	0.576 (0.504, 0.648)
Dataset 1	Dataset 4, Day -3	BSS	0.006 (0, 0.035)	1 (0.964, 1)	1 (0.025, 1)	0.395 (0.334, 0.457)	0.576 (0.504, 0.648)
Dataset 1	Dataset 4, Day -1	Original	0.936 (0.885, 0.969)	0.446 (0.347, 0.548)	0.723 (0.656, 0.783)	0.818 (0.691, 0.909)	0.868 (0.823, 0.913)
Dataset 1	Dataset 4, Day -1	Full	0.006 (0, 0.035)	1 (0.964, 1)	1 (0.025, 1)	0.395 (0.334, 0.457)	0.551 (0.479, 0.623)
Dataset 1	Dataset 4, Day -1	BSS	0.006 (0, 0.035)	1 (0.964, 1)	1 (0.025, 1)	0.395 (0.334, 0.457)	0.554 (0.482, 0.627)
Dataset 3	Dataset 3	Original	0.806 (0.727, 0.87)	0.835 (0.806, 0.862)	0.464 (0.398, 0.532)	0.961 (0.942, 0.974)	0.88 (0.85, 0.91)
Dataset 3	Dataset 3	Full	0.76 (0.677, 0.831)	0.85 (0.822, 0.876)	0.473 (0.404, 0.544)	0.952 (0.933, 0.967)	0.9 (0.87, 0.92)
Dataset 3	Dataset 3	BSS	0.752 (0.668, 0.824)	0.879 (0.853, 0.902)	0.524 (0.45, 0.598)	0.952 (0.934, 0.967)	0.88 (0.85, 0.91)
Dataset 3	Dataset 1	Original	0.572 (0.548, 0.595)	0.854 (0.842, 0.865)	0.622 (0.598, 0.646)	0.825 (0.814, 0.837)	0.793 (0.78, 0.806)
Dataset 3	Dataset 1	Full	0.063 (0.052, 0.076)	0.995 (0.992, 0.997)	0.836 (0.76, 0.895)	0.716 (0.704, 0.728)	0.79 (0.777, 0.803)
Dataset 3	Dataset 1	BSS	0.004 (0.001, 0.008)	1 (0.998, 1)	0.75 (0.349, 0.968)	0.704 (0.692, 0.716)	0.722 (0.707, 0.737)
Dataset 3	Dataset 4, Day -3	Original	0.538 (0.457, 0.618)	0.772 (0.678, 0.85)	0.785 (0.695, 0.859)	0.52 (0.437, 0.602)	0.739 (0.677, 0.8)
Dataset 3	Dataset 4, Day -3	Full	0.41 (0.332, 0.492)	0.762 (0.667, 0.841)	0.727 (0.622, 0.817)	0.456 (0.379, 0.534)	0.658 (0.587, 0.729)
Dataset 3	Dataset 4, Day -3	BSS	0.256 (0.19, 0.332)	0.772 (0.678, 0.85)	0.635 (0.504, 0.753)	0.402 (0.332, 0.475)	0.57 (0.497, 0.643)
Dataset 3	Dataset 4, Day -1	Original	0.853 (0.787, 0.904)	0.614 (0.512, 0.709)	0.773 (0.703, 0.834)	0.729 (0.622, 0.82)	0.823 (0.771, 0.874)
Dataset 3	Dataset 4, Day -1	Full	0.878 (0.816, 0.925)	0.554 (0.452, 0.653)	0.753 (0.683, 0.814)	0.747 (0.633, 0.84)	0.742 (0.676, 0.808)
Dataset 3	Dataset 4, Day -1	BSS	0.731 (0.654, 0.799)	0.584 (0.482, 0.681)	0.731 (0.654, 0.799)	0.584 (0.482, 0.681)	0.681 (0.61, 0.751)
Dataset 4, Day -3	Dataset 4, Day -3	Original	0.814 (0.744, 0.872)	0.723 (0.625, 0.807)	0.819 (0.75, 0.876)	0.716 (0.618, 0.801)	0.83 (0.77, 0.88)
Dataset 4, Day -3	Dataset 4, Day -3	Full	0.827 (0.758, 0.883)	0.782 (0.689, 0.858)	0.854 (0.788, 0.906)	0.745 (0.651, 0.825)	0.85 (0.8, 0.9)
Dataset 4, Day -3	Dataset 4, Day -3	BSS	0.814 (0.744, 0.872)	0.792 (0.7, 0.866)	0.858 (0.791, 0.91)	0.734 (0.641, 0.814)	0.86 (0.81, 0.91)
Dataset 4, Day -3	Dataset 1	Original	0.699 (0.676, 0.72)	0.793 (0.78, 0.805)	0.587 (0.565, 0.609)	0.862 (0.85, 0.873)	0.821 (0.809, 0.833)
Dataset 4, Day -3	Dataset 1	Full	0.881 (0.864, 0.896)	0.553 (0.537, 0.568)	0.454 (0.436, 0.471)	0.917 (0.905, 0.927)	0.836 (0.824, 0.847)
Dataset 4, Day -3	Dataset 1	BSS	0.875 (0.859, 0.891)	0.562 (0.546, 0.577)	0.457 (0.44, 0.474)	0.914 (0.903, 0.925)	0.838 (0.827, 0.849)
Dataset 4, Day -3	Dataset 3	Original	0.938 (0.881, 0.973)	0.257 (0.225, 0.29)	0.183 (0.154, 0.214)	0.959 (0.921, 0.982)	0.585 (0.544, 0.627)
Dataset 4, Day -3	Dataset 3	Full	0.922 (0.862, 0.962)	0.372 (0.337, 0.408)	0.206 (0.174, 0.242)	0.964 (0.936, 0.983)	0.698 (0.654, 0.741)
Dataset 4, Day -3	Dataset 3	BSS	0.922 (0.862, 0.962)	0.353 (0.318, 0.388)	0.201 (0.17, 0.236)	0.963 (0.932, 0.982)	0.687 (0.643, 0.731)
Dataset 4, Day -3	Dataset 3 (Age < 16)	Original	0.708 (0.489, 0.874)	0.747 (0.674, 0.811)	0.288 (0.178, 0.421)	0.947 (0.893, 0.978)	0.826 (0.742, 0.91)
Dataset 4, Day -3	Dataset 3 (Age < 16)	Full	0.75 (0.533, 0.902)	0.711 (0.636, 0.778)	0.273 (0.17, 0.396)	0.952 (0.898, 0.982)	0.806 (0.725, 0.888)
Dataset 4, Day -3	Dataset 3 (Age < 16)	BSS	0.75 (0.533, 0.902)	0.705 (0.629, 0.773)	0.269 (0.168, 0.391)	0.951 (0.897, 0.982)	0.791 (0.707, 0.875)
Dataset 4, Day -1	Dataset 4, Day -1	Original	0.827 (0.758, 0.883)	0.752 (0.657, 0.833)	0.838 (0.77, 0.892)	0.738 (0.642, 0.82)	0.86 (0.81, 0.91)
Dataset 4, Day -1	Dataset 4, Day -1	Full	0.801 (0.73, 0.861)	0.792 (0.7, 0.866)	0.856 (0.789, 0.909)	0.721 (0.628, 0.802)	0.88 (0.84, 0.93)
Dataset 4, Day -1	Dataset 4, Day -1	BSS	0.814 (0.744, 0.872)	0.802 (0.711, 0.875)	0.864 (0.798, 0.915)	0.736 (0.644, 0.816)	0.88 (0.84, 0.93)
Dataset 4, Day -1	Dataset 1	Original	0.424 (0.401, 0.448)	0.951 (0.944, 0.957)	0.785 (0.757, 0.811)	0.797 (0.785, 0.808)	0.828 (0.816, 0.84)
Dataset 4, Day -1	Dataset 1	Full	0.58 (0.556, 0.603)	0.892 (0.882, 0.901)	0.693 (0.668, 0.717)	0.834 (0.823, 0.845)	0.829 (0.817, 0.84)
Dataset 4, Day -1	Dataset 1	BSS	0.586 (0.562, 0.61)	0.899 (0.89, 0.909)	0.711 (0.686, 0.734)	0.838 (0.826, 0.849)	0.832 (0.82, 0.843)
Dataset 4, Day -1	Dataset 3	Original	0.891 (0.825, 0.939)	0.523 (0.486, 0.559)	0.248 (0.21, 0.29)	0.965 (0.941, 0.98)	0.718 (0.68, 0.756)
Dataset 4, Day -1	Dataset 3	Full	0.775 (0.693, 0.844)	0.649 (0.613, 0.684)	0.281 (0.235, 0.331)	0.942 (0.918, 0.961)	0.768 (0.73, 0.807)
Dataset 4, Day -1	Dataset 3	BSS	0.822 (0.745, 0.883)	0.61 (0.574, 0.646)	0.272 (0.228, 0.319)	0.951 (0.927, 0.969)	0.752 (0.713, 0.791)
Dataset 4, Day -1	Dataset 3 (Age < 16)	Original	0.667 (0.447, 0.844)	0.904 (0.848, 0.944)	0.5 (0.319, 0.681)	0.949 (0.903, 0.978)	0.87 (0.796, 0.945)
Dataset 4, Day -1	Dataset 3 (Age < 16)	Full	0.667 (0.447, 0.844)	0.861 (0.799, 0.91)	0.41 (0.256, 0.579)	0.947 (0.898, 0.977)	0.837 (0.759, 0.915)
Dataset 4, Day -1	Dataset 3 (Age < 16)	BSS	0.625 (0.406, 0.812)	0.855 (0.793, 0.905)	0.385 (0.234, 0.554)	0.94 (0.89, 0.972)	0.839 (0.76, 0.917)
