# Supplementary figures and images for: Assessing generalizability of a dengue classifier across multiple datasets
Source: PLoS One. 2025 Jun 3;20(6):e0323886. doi: 10.1371/journal.pone.0323886 (PMC12132959; doi:10.1371/journal.pone.0323886)

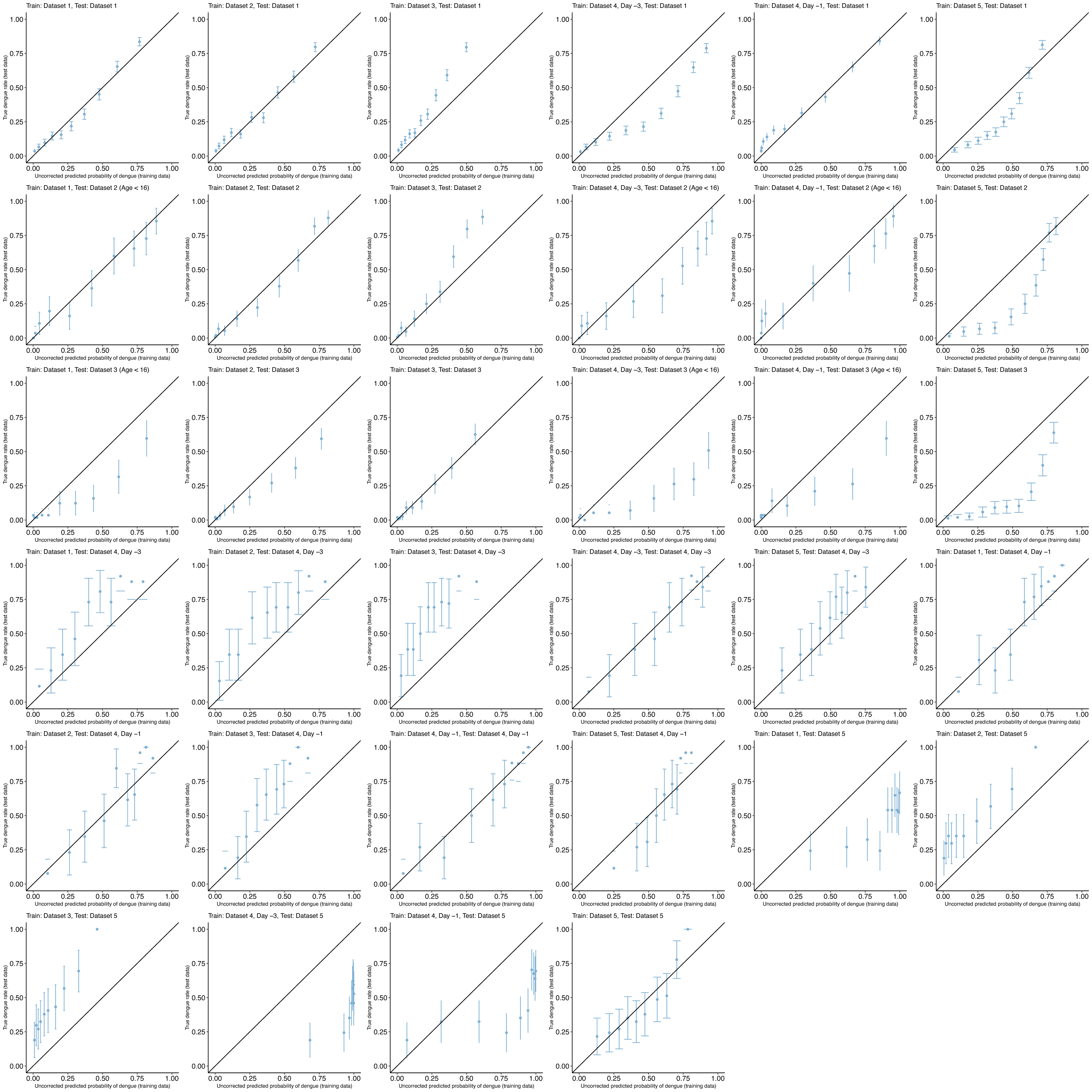

Supplement: S1 Fig — The logistic regression model is fit on the training set, and applied to the test set to calculate predicted probabilities. These raw predicted probabilities are compared against true instances of dengue. (TIF) [file pone.0323886.s007.tif]

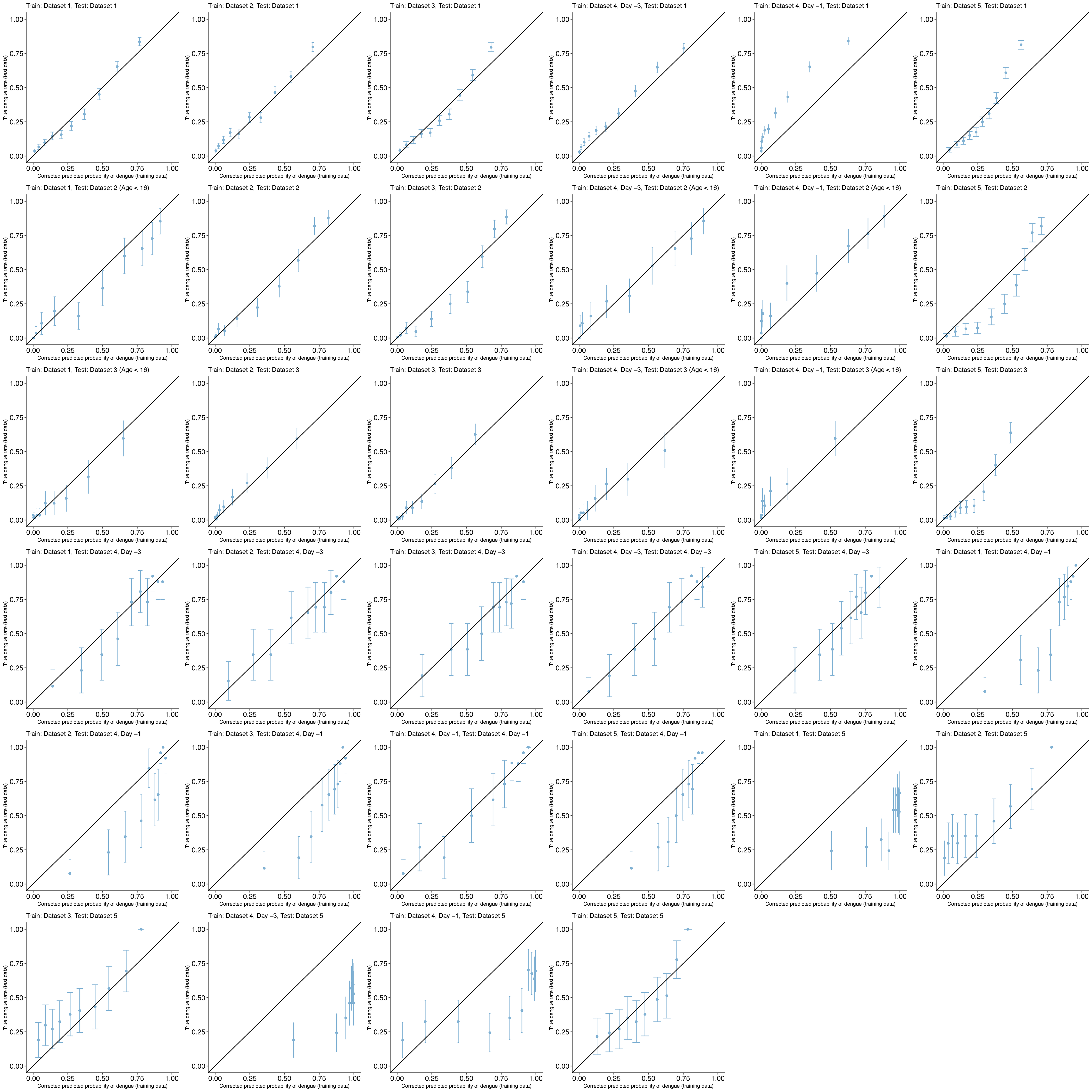

Supplement: S2 Fig — The logistic regression model is fit on the training set, and applied to the test set to calculate predicted probabilities. The predicted probabilities are adjusted with a label shift correction to account for a different rate of dengue fever in the test dataset (see Eq 1 in the manuscript). (TIF) [file pone.0323886.s008.tif]

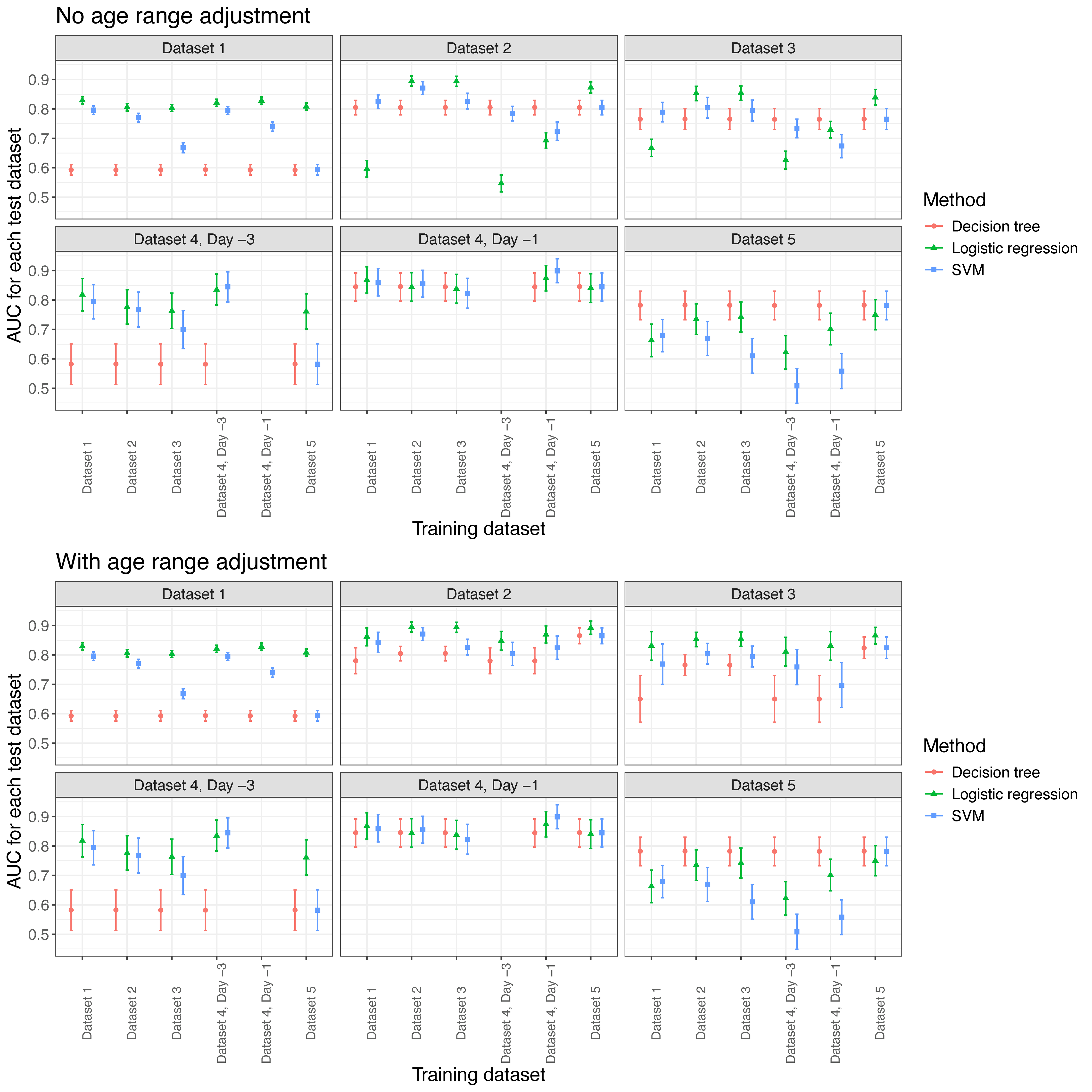

Supplement: S3 Fig — Recall that the original explanatory variables are Age, WBC, and PLT (see Table 1 in the main manuscript). Each panel represents one test set, with the predictive performance on that test set (the AUC) displayed for each training set. Performance is shown with and without age range restrictions. Without age range restrictions, all complete cases in the training and test datasets are used. With age range restrictions, the test dataset is restricted (if possible) to match the age range of the training data (<16 if training data is Dataset 1 or Dataset 4 and test data is Dataset 2 or Dataset 3; >16 if training data is Dataset 5 and test data is Dataset 2 or Dataset 3). (TIF) [file pone.0323886.s009.tif]

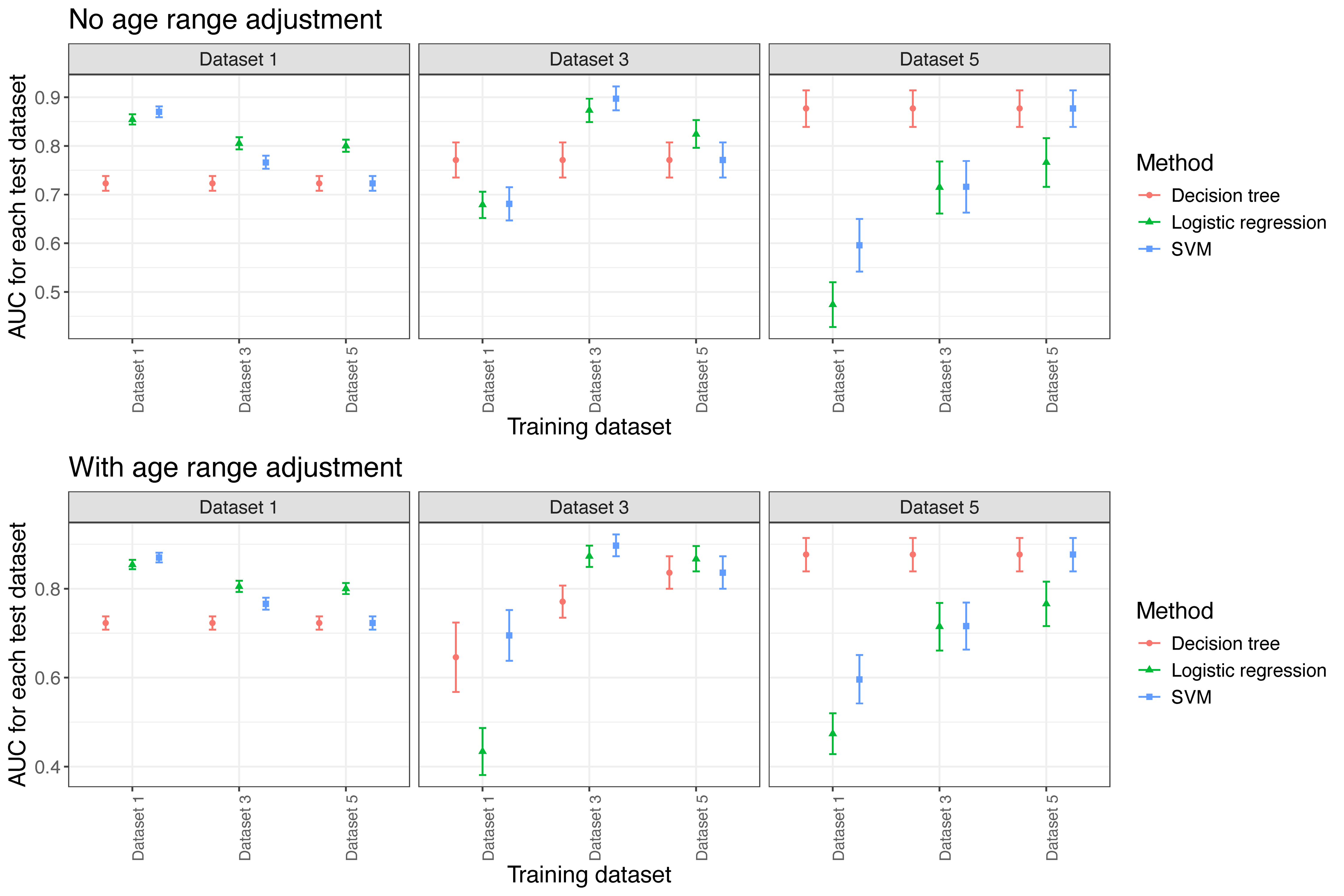

Supplement: S4 Fig — The variables in Alternative Subset 1 can be found in Table 1. Each panel represents one test set, with the predictive performance on that test set (the AUC) displayed for each training set. Performance is shown with and without age range restrictions. Without age range restrictions, all complete cases in the training and test datasets are used. With age range restrictions, the test dataset is restricted (if possible) to match the age range of the training data (<16 if training data is Dataset 1 or Dataset 4 and test data is Dataset 2 or Dataset 3; >16 if training data is Dataset 5 and test data is Dataset 2 or Dataset 3). (TIF) [file pone.0323886.s010.tif]

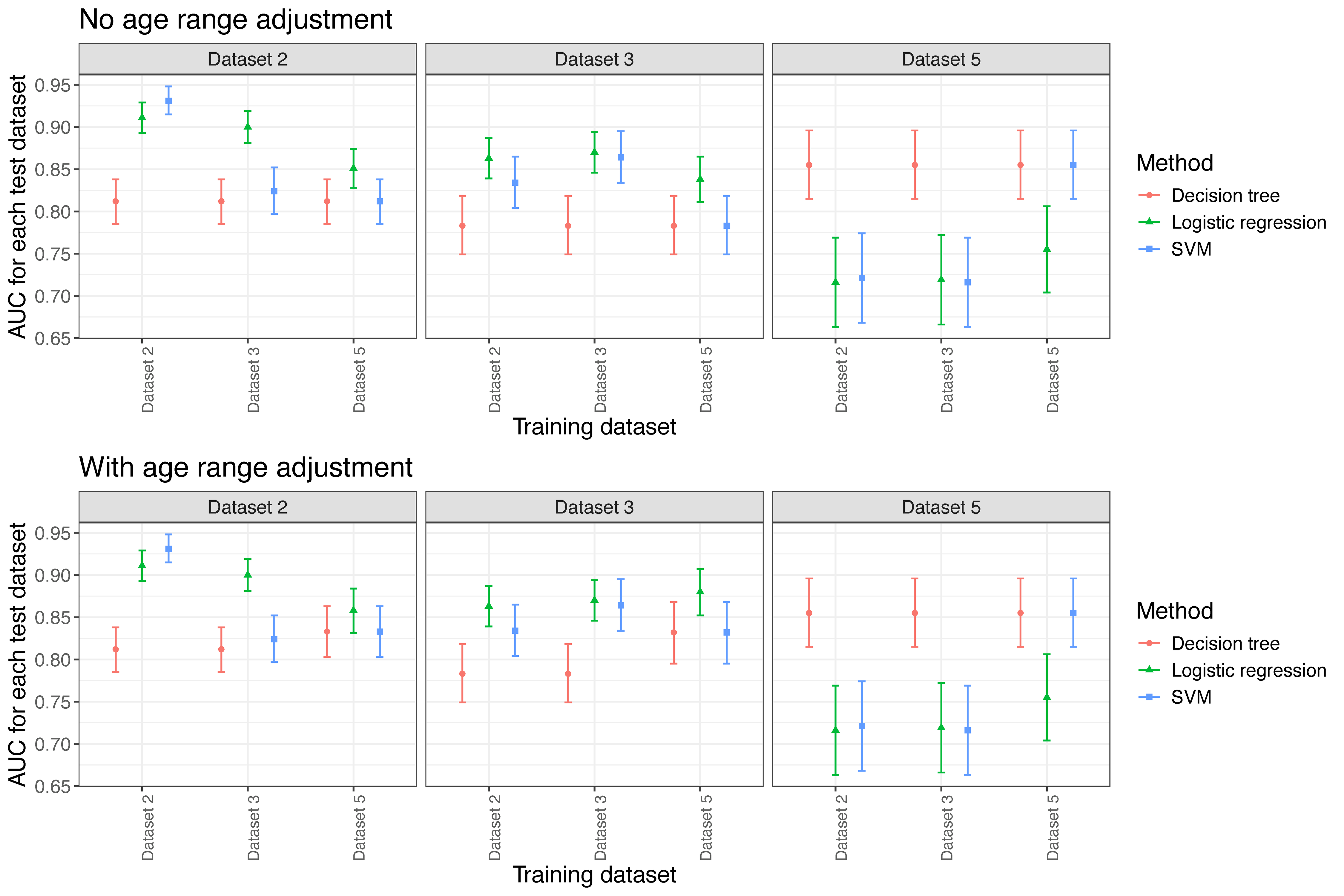

Supplement: S5 Fig — The variables in Alternative Subset 2 can be found in Table 1. Each panel represents one test set, with the predictive performance on that test set (the AUC) displayed for each training set. Performance is shown with and without age range restrictions. Without age range restrictions, all complete cases in the training and test datasets are used. With age range restrictions, the test dataset is restricted (if possible) to match the age range of the training data (<16 if training data is Dataset 1 or Dataset 4 and test data is Dataset 2 or Dataset 3; >16 if training data is Dataset 5 and test data is Dataset 2 or Dataset 3). (TIF) [file pone.0323886.s011.tif]

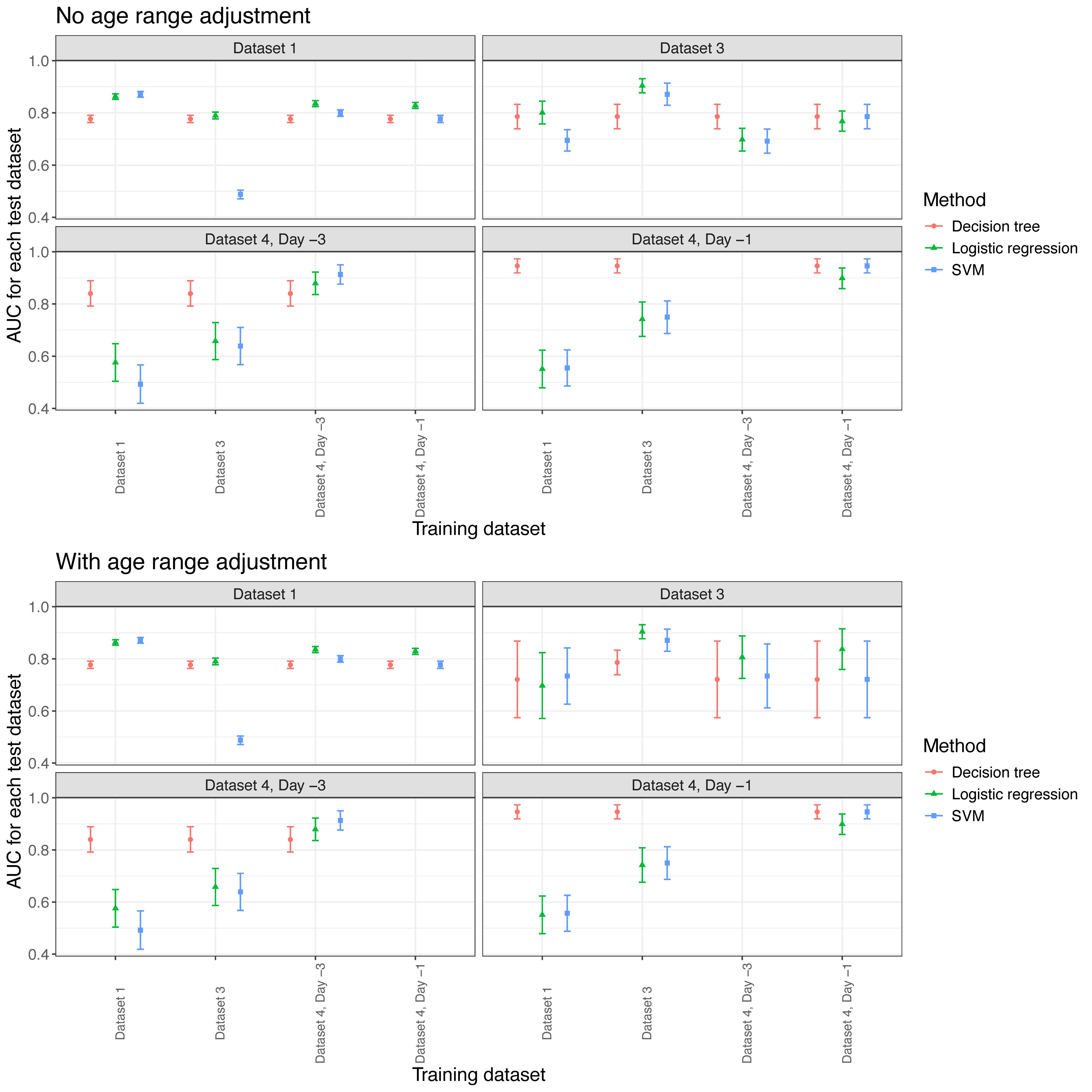

Supplement: S6 Fig — The variables in Alternative Subset 3 can be found in Table 1. Each panel represents one test set, with the predictive performance on that test set (the AUC) displayed for each training set. Performance is shown with and without age range restrictions. Without age range restrictions, all complete cases in the training and test datasets are used. With age range restrictions, the test dataset is restricted (if possible) to match the age range of the training data (<16 if training data is Dataset 1 or Dataset 4 and test data is Dataset 2 or Dataset 3; >16 if training data is Dataset 5 and test data is Dataset 2 or Dataset 3). (TIF) [file pone.0323886.s012.tif]
